# Contextual determinants associated with children’s and adolescents’ mental health care utilization: a systematic review

**DOI:** 10.1007/s00787-022-02077-5

**Published:** 2022-09-21

**Authors:** S. Verhoog, D. G. M. Eijgermans, Y. Fang, W. M. Bramer, H. Raat, W. Jansen

**Affiliations:** 1 Department of Public Health, Erasmus MC, University Medical Centre, P.O. box 2040, 3000 CA Rotterdam, The Netherlands; 2 The Generation R Study Group, Erasmus MC, University Medical Centre, Rotterdam, the Netherlands; 3 Medical Library, Erasmus MC, University Medical Centre, Rotterdam, The Netherlands; 4 Department of Social Development, City of Rotterdam, Rotterdam, the Netherlands

**Keywords:** Patient acceptance of health care, Social class, Urban population, Child, Adolescent, Psychotherapy

## Abstract

Determinants at the contextual level are important for children’s and adolescents’ mental health care utilization, as this is the level where policy makers and care providers can intervene to improve access to and provision of care. The objective of this review was to summarize the evidence on contextual determinants associated with mental health care utilization in children and adolescents. A systematic literature search in five electronic databases was conducted in August 2021 and retrieved 6439 unique records. Based on eight inclusion criteria, 74 studies were included. Most studies were rated as high quality (79.7%) and adjusted for mental health problems (66.2%). The determinants that were identified were categorized into four levels: organizational, community, public policy or macro-environmental. There was evidence of a positive association between mental health care utilization and having access to a school-based health center, region of residence, living in an urban area, living in an area with high accessibility of mental health care, living in an area with high socio-economic status, having a mental health parity law, a mental health screening program, fee-for-service plan (compared to managed care plan), extension of health insurance coverage and collaboration between organizations providing care. For the other 35 determinants, only limited evidence was available. To conclude, this systematic review identifies ten contextual determinants of children’s and adolescents’ mental health care utilization, which can be influenced by policymakers and care providers. Implications and future directions for research are discussed

PROSPERO ID: CRD42021276033.

**Supplementary Information:**

The online version contains supplementary material available at 10.1007/s00787-022-02077-5.

## Introduction

Childhood and adolescence are critical phases in life for mental health. The onset of a first mental disorder occurs before age 14 in one-third of individuals, age 18 in almost half, and before age 25 in more than half of individuals [1]. A recent systematic review of high-income countries shows an overall prevalence of any childhood mental disorder of 13% [2]. The consequences of mental disorders include a negative impact on quality of life [3] and the development of school careers [4–6]. These disorders can even continue into adulthood [7] and may be related to worse employment outcomes [8, 9]. Apart from the individual burden of mental disorders for children and adolescents, there is also a collective social and economic burden [10, 11]. For instance, children with mental disorders may be more often involved in crime, have extra educational needs or are more likely to end up in foster care or residential care compared to children without mental disorders [12]. Despite the high prevalence and negative consequences of mental disorders in children and adolescents, many do not receive any services to deal with or reduce these disorders [2, 13, 14].

Effective prevention and early intervention strategies for mental disorders have the potential to significantly reduce the burden of disease and improve an individual’s quality of life [15, 16]. However, to develop such strategies, it is crucial to properly understand which factors contribute to children’s and adolescents’ service use. Andersen et al. [17] created the Behavioural Model of Health Service use, a theoretical framework of health service use including individual and contextual determinants. Associations of mental health service use with determinants at the individual level such as age, gender, ethnicity, and family situation have been summarized in earlier systematic reviews. For example, Elster et al. [18] performed a review on racial and ethnic disparities in care utilization among adolescents and the review by Ryan et al. [19] focused on family-related factors in associations with mental health service use. Besides determinants on the individual and family level, determinants on broader contextual levels have been shown to also explain mental health service use.

However, summarized evidence on contextual determinants of mental health service use, such as health organization and provider-related factors, is scarce and the systematic reviews available have very specific topics or study populations. The systematic review by Werlen et al. [20] is limited to interventions to improve children's access to mental health care at both the individual and contextual level. The systematic review by So et al. summarizes the evidence on policy levers to promote access to and utilization of children’s mental health services, limited to the United States of America (U.S.A.) [21]. The systematic review by Eijgermans et al. summarizes individual-level and contextual determinants associated with mental health care utilization [22]. However, this review solely included longitudinal, population-based studies.

This systematic review aims to identify, summarize and discuss all available evidence from studies on contextual determinants of mental health care utilization in children and adolescents. In this review, mental health care is defined as inpatient and outpatient services and medication use to treat mental, behavioral and emotional problems. Contextual determinants are grouped according to McLeroy’s ecological model [23]. This is a conceptual framework that distinguishes different layers surrounding an individual. In our review, we focus on layers beyond the individual and family level. These layers consist of contextual determinants that are beyond individual control, and influence multiple unrelated children, such as the school or the community, and more distal public policy. The last and most distant layer comprises macro-environmental determinants that are more difficult to influence, e.g. climate. Many contextual determinants can be influenced by or are the responsibility of policymakers and care providers. Insight into which contextual determinants influence mental health care utilization will enable care providers and policy makers to improve access to and provision of care and decrease treatment gaps in their populations. Furthermore, this knowledge can contribute to the development of preventive strategies and give directions for future research on this topic.

## Methods

### Literature search

This review was conducted and reported in accordance with the PRISMA statement [24] and was registered at PROSPERO (ID: CRD42021276033) on August 30, 2021. An experienced information specialist from Erasmus Medical Centre (WB) created and performed the search in five databases (Embase via Embase.com, MEDLINE ALL via Ovid, Web of Science Core Collection, the Cochrane Library CENTRAL register of Trials via Wiley and PsycINFO via Ovid). Studies examining the association between any contextual factor and children’s and adolescent’s mental health care utilization were identified from database inception until August 31, 2021 (date last searched). To capture all relevant studies, we deliberately used a broad search strategy using terms related to mental health care or psychotherapy, children or adolescents, health care utilization or therapy enrollment, and study types (controlled, cohort and international studies). We did not apply any restrictions on date but excluded non-English language articles and conference abstracts. The search can be found in Supplement 1. The search is similar to the search of a previous systematic review [22]. However, different study selection criteria were applied in both reviews. In contrast to the previous systematic review, in the current systematic review, we did not restrict the selection criteria to longitudinal, population-based studies. To identify additional relevant studies, we checked the reference lists of the studies included in the current review and other relevant systematic reviews. All articles were imported in EndNote and de-duplicated with the method by Bramer et al. [25].

### Study selection criteria

For inclusion in the review, studies had to meet all of the following selection criteria:

(i) Language: the full text is written in English;
(ii) Study type: the study is quantitative, empirical, peer-reviewed, and published in a scientific journal;
(iii) Study population: the study population is children under the age of 18 years, or a population with a mean age under 18 years and no participants over the age of 21;
(iv) Geographic region: the study population is living in a Western geographical region, this to increase the comparability among studies in an already broad systematic review. Contextual determinants might be different in non-Western countries [26]. Western geographical region includes Europe (except Turkey), Northern America (the U.S.A. and Canada) and Oceania (Australia and New Zealand);
(v) Outcome: the outcome of the study has to be mental health care defined as inpatient and outpatient services and medication use for the treatment of mental, behavioral and emotional problems;
(vi) Determinant: the determinant has to be a contextual factor that is beyond individual control, and has an influence on multiple unrelated children. All individual or family-related determinants were excluded;
(vii) Comparison group: a comparison group or reference group using no care has to be part of the study;
(viii) Statistical analysis: a statistical analysis assessing an association must be performed.

Using these criteria, two reviewers (SV and DE) independently screened titles and abstracts for eligibility. In case of disagreement, a decision was made by consensus or the study was included for full-text evaluation. Hereafter, two reviewers (SV and DE) assessed the full-text of the remaining studies with the same criteria. During the full-text evaluation, there were seven cases of disagreement, for which a third reviewer (WJ) was consulted.

### Data extraction

Using a predesigned form, the following data were extracted as characteristics of the included studies: first author, year of publication, country, study design, number of participants, database, type of study population, age, type of mental health care and reporter of mental health care utilization. Regarding the studied determinants, the following data were extracted: the determinant studied in association with mental health care utilization, whether this association was significant and if so, the direction and measure of the association, and if applicable, any subgroup findings and whether or not the association was adjusted for mental health problems. This process was completed by one researcher (SV). A random selection of 8 studies (10%) was assessed by a second researcher (DE) with almost perfect inter-rater agreement (Kappa = 0.88) [27].

### Quality assessment

For quality assessment of the included studies, the QualSyst tool of Kmet et al. [28] was used. In this checklist, 14 items are listed, which were scored with 2 (yes), 1 (partial), or 0 points (no) each. To calculate the quality score per study, all scores were added together and divided by the total possible sum. This process was completed by one researcher (DE). A random selection of 8 studies (10%) was assessed by a second researcher (SV) with almost perfect inter-rater agreement (Kappa = 0.86) [27]. Based on this final score, the quality of the study was rated high (≥ 0.80), medium (≥ 0.60 and < 0.80), or low (< 0.60). These cutoffs are in line with other studies that used the QualSyst Tool [29–31].

### Data synthesis

**Fig. 1:**
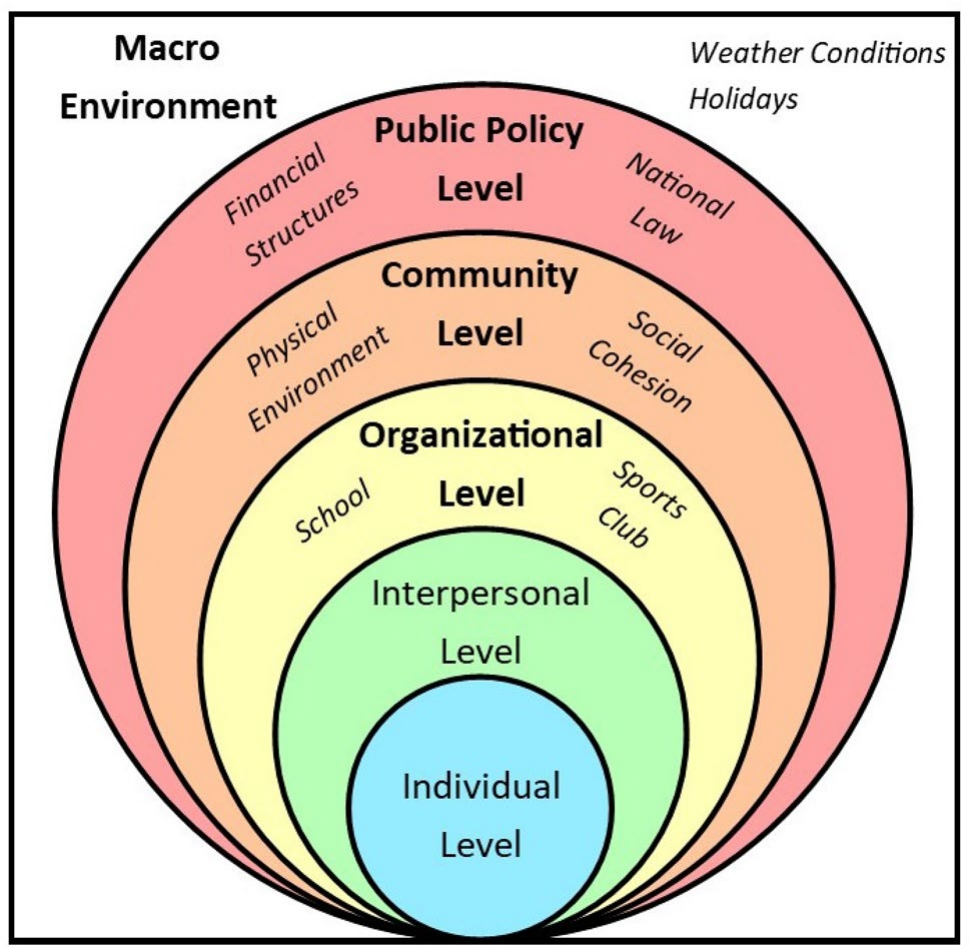
Modified version of McLeroy’s ecological model, indicating the different layers of an individual’s context. The layers in bold are studied in this review

Identified determinants were categorized according to a slightly modified version of McLeroy’s widely used ecological model, comprising three contextual levels surrounding an individual, beyond the individual and interpersonal level (Fig. 1) [23]. The first is the organizational level, including determinants that are related to institutions visited by an individual, such as school or a sports club. The second is the community level, including determinants that are related to the community and neighborhood where an individual lives, such as its physical environment, availability of services or the quality of relationships within the neighborhood. The third is the public policy level, including determinants that are related to national and/or regional policies and the organization of care. Examples are national or state-wide laws, financial structures for the provision of care and the extent of collaboration between health care providers. For the purpose of this review, a fourth level was added. This level is the macro-environmental, including determinants that are difficult to control, such as weather conditions, seasons and national Holidays.

To summarize the evidence on the association of contextual determinants and children’s and adolescents’ mental health care utilization, a previously established method was used [32–34]. The number of studies that reported a significant association in the same direction between a contextual determinant and mental health care utilization was divided by the total number of studies that examined that determinant. Determinants investigated by four or more studies were coded as: no association (00) when 0–33% of studies found a significant association in the same direction; inconsistent association (??) when 34–59% of studies found a significant association in the same direction; positive (+ +) or negative (− −) association when 60–100% of studies found a significant association in the same direction. Determinants investigated by three studies or less were coded as limited evidence. To provide insight in the direction of the limited evidence, the same criteria were applied to the determinants investigated by four or more studies, but only with one symbol (i.e., 0, ?, + and −). The rules for classifying the level of evidence are summarized in Table 1. There are three notes to this classification: 1) if multiple studies from one database assessed the same contextual determinant, this counts as one study in summarizing the evidence; 2) if a study reports both an association and no association (e.g., when the determinant is associated with outpatient care, but not with inpatient), the association outweighs no association; and 3) if the number of studies reporting a positive and negative association was equal, and based on above rules would be labelled as evidence of a positive or negative association, the determinant was nevertheless coded as inconsistent evidence.

**Table 1:** Rules for classifying the level of evidence

% of studies reporting a significant association	Summary code	Meaning of summary code
0–33	00	No association
34–59	??	Inconsistent evidence
60–100	+ + – –	Positive association Negative association

When three or less studies reported an association or no association, it was coded as limited evidence. To provide insight in the direction of the association from three or less, only one symbol was assigned, i.e. 0, ?, + or –

## Results

### Study identification and selection

**Fig. 2:**
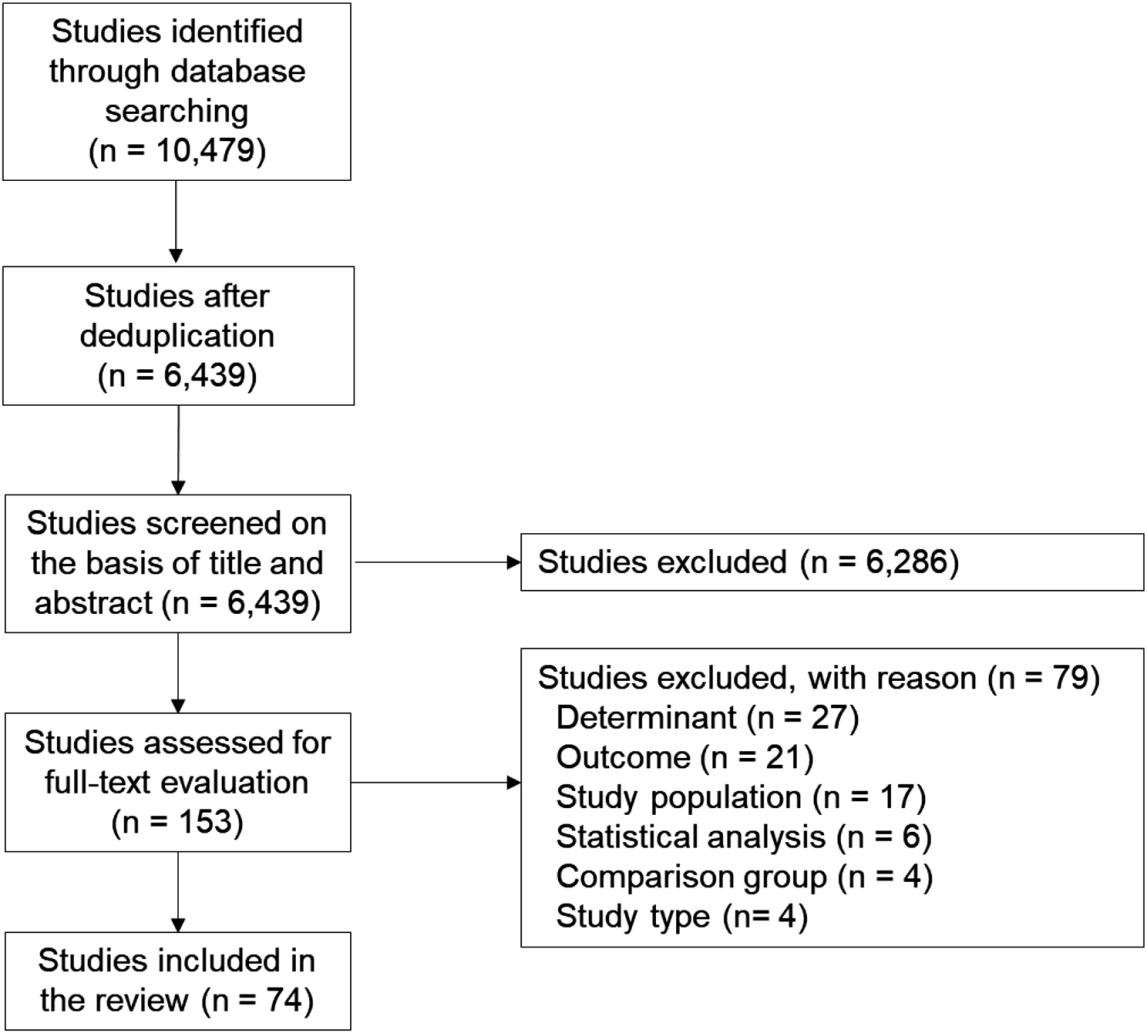
Flowchart of study selection

The flowchart of study selection is presented in Fig. 2. In total, 10,479 potentially relevant studies were identified. After de-duplication, 6439 studies were screened on title and abstract, which resulted in 153 eligible studies for full-text screening. Of those, 74 studies met the selection criteria and were included in the review [35–108].

### Characteristics of the included studies

The characteristics of the included studies are summarized in Table 2. The majority of the included studies were conducted in the U.S.A. (83.8%) and were published in 2010 or later (63.5%). In addition, 71.3% of the included studies were conducted with a longitudinal, experimental or quasi-experimental design. The number of participants varied widely, ranging from fewer than 100 participants to more than 1,000,000 participants. No studies were conducted solely among children in early childhood (0–3 years old) and more than half of the studies (60.8%) included a broad age range covering more than one age category (e.g., childhood and adolescence). Approximately one-third of the included studies (35.1%) were performed in a general population, the other studies were performed in specific populations such as children with mental health problems or using any type of care (35.1%), children from low-income families (14.9%), children involved in the child welfare system (4.1%), children with disabilities (2.7%) or a combination of these specific groups (5.4%). Various types of mental health care were assessed in the included studies, mostly a combination of inpatient and outpatient (33.8%) or outpatient only (27.0%), whereby half of the studies (50.0%) used administrative data. Approximately two-thirds (66.2%) of the studies adjusted for (the level of) mental health problems in the studied associations. Detailed information on the characteristics of the included studies can be found in Supplement 2.

**Table 2:** Characteristics of the included studies (*N* = 74)

Characteristics	N of studies (%)^a^
Place of study	
Europe	10 (13.5)
Northern America	62 (83.8)
Oceania	2 (2.7)
Year published	
≥ 2020	10 (13.5)
2010–2019	37 (50.0)
2000–2009	21 (28.4)
< 2000	6 (8.1)
Study design	
Case–control study	3 (4.1)
Randomized study	5 (6.8)
Quasi-experimental study	17 (23.0)
Longitudinal/cohort study	30 (40.5)
Cross sectional study	19 (25.7)
Number of participants	
< 100	1 (1.4)
100–999	17 (23.0)
1000–9999	25 (33.8)
10,000–99,999	17 (23.0)
100,000–999,999	6 (8.1)
≥ 1,000,000	5 (6.8)
Other (e.g. person years)	3 (4.1)
Age children	
Early childhood (0–3 years)	0 (0.0)
Childhood (± 4–12 years)^b^	10 (13.5)
Adolescence (± 13–21 years)^b^	19 (25.7)
More than one age group	45 (60.8)
Type of study population	
General population	26 (35.1)
With mental health problems/care	26 (35.1)
Low income	11 (14.9)
Low income and mental health problems/care	4 (5.4)
Involved in child welfare	3 (4.1)
With disabilities	2 (2.7)
Other	2 (2.7)
Type of mental health care	
Outpatient	20 (27.0)
Inpatient	4 (5.4)
Medication use	2 (2.7)
Outpatient and inpatient	25 (33.8)
Outpatient and medication use	3 (4.1)
Inpatient and medication use	0 (0.0)
Outpatient, inpatient and medication use	10 (13.5)
Not specified	10 (13.5)
Reporter of mental health care utilization	
Administrative data	37 (50.0)
Caregiver	25 (33.8)
Self-reported by youth	8 (10.8)
Agency's research staff	1 (1.4)
Self-report + caregiver	2 (2.7)
Administrative data + caregiver	1 (1.4)
Adjustment for mental health problems	
Yes	49 (66.2)
No	24 (32.4)
Partial	1 (1.4)
Quality of studies (QualSyst Tool)	
High (≥ 0.80)	59 (79.7)
Middle (≥ 0.60 and < 0.80)	15 (20.3)
Low (< 0.60)	0 (0.0)

a) Due to rounding, the percentages might not add up to 100%; b) The age range in the included studies was allowed to differ with a maximum of two years to be included in one of the categories

### Quality assessment

Most studies (79.7%) were rated as high quality, the remaining studies were rated as moderate quality. No studies received a low-quality score. The items “description of the study design” and “method of subject selection” most often scored poorly. Detailed information on the quality assessment can be found in Supplement 3.

### Contextual determinants associated with mental health care utilization

Table 3 provides an overview of all contextual determinants of mental health care utilization in children and adolescents that were investigated in the 74 studies. Studies that were both rated as high quality and adjusted for mental health problems are presented in bold, hereafter referred to as high-quality well-adjusted studies. In total, 45 determinants were identified, of which 35 with limited evidence (77.7%). Results are presented according to levels of McLeroy’s modified ecological model: organizational, community, public policy and macro-environmental (Fig. 1) [23].

**Table 3:** Evidence of the 74 included studies on the association between contextual determinants and mental health care utilization among children and adolescents

Determinant	Studies reporting negative association	Studies reporting no association	Studies reporting positive association	*n*/*N*^a^	Summary^b^
*Organizational level*					
Access to school-based health centre		Kaplan, 1999; Slade, 2002	Hussaini, 2021; **Hutchington, 2012**; Kaplan, 1998; Slade, 2002; Williams, 2015	5/6	+ +
School mental health resources	**Green, 2013**	**Green, 2013**	**Green, 2013**	1/1	?
Academic performance		**Green, 2013**; **Kang-Yi, 2013**	**Green, 2013**	1/2	?
Emotional/behavioral problems at school		**Green, 2013**		0/1	0
Extent of collaboration with families	**Green, 2013**	**Green, 2013**		1/1	–
Monthly student absent days		**Kang-Yi, 2013**		0/1	0
School ethnic composition		**Green, 2013**		0/1	0
School location (county vs city)	**Green, 2013**	Britto, 2001; **Green, 2013**		1/2	?
School SES	**Halladay, 2020**	**Halladay, 2020**		1/1	–
School size		**Halladay, 2020**		0/1	0
School type (public vs private)	**Green, 2013**	**Green, 2013**		1/1	–
Student/teacher ratio		**Green, 2013**	**Green, 2013**	1/1	+
Teacher engagement		**Green, 2013; Halladay, 2020; Kang-Yi, 2013**	**Green, 2013**; **Halladay, 2020**	2/3	+
Educational intervention about ADHD for teachers		Sayal, 2010		0/1	0
Links-to-learning intervention			Atkins, 2015	1/1	+
Multi-dimensional school-based intervention		Britto, 2001; **Kang-Yi, 2013**		0/2	0
*Community level*					
Region of residence^d^	NA	**Abbas, 2017**; **Bryson, 2015**; **Cook, 2004**; Davila, 2020; Patrick, 1993; Sturm, 2003; Zablotsky, 2019;	**Abbas, 2017**; **Bird, 2008**; **Bryson, 2015**; **Cook, 2004**; Davila, 2020; **Howell, 2008**; Sturm, 2003; **Waxmonsky, 2019**	8/10	+ +
Accessibility of services (high vs low)	**Bai, 2009**	**Cummings, 2014**; **Hurlburt 2004**; **Raghavan, 2006;** Slade, 2002; Patrick, 1993	**Abbas, 2017**; **Bai, 2009**; **Kovess-Masfety, 2017**; **Raghavan, 2006**; Slade, 2002	5/8	+ +
Area level socio-economic status (high vs low)	van der Linden, 2003	**Cummings, 2014**; **Hurlburt 2004**; Ivert, 2013	**Abbas, 2017**; **Efron, 2019**; **Fitts, 2019**; **Johnson, 2016**; Kim, 2018	5/9	+ + ^c^
Living in an honor state	Brown, 2014			1/1	–
Income inequality (high vs low)	Finnvold, 2019		Finnvold, 2019	1/1	?
Urbanicity (urban vs rural)	**Brannan, 2005**^**e**^**;** Mendenhall, 2012	**Cook, 2004**; **Johnson, 2016**; Patrick, 1993; Slade, 2002; Sullivan, 2015	**Bai, 2009**; **Brannan 2005**^**e**^; **Bryson, 2015**; **Cohen, 1993**; **Cook, 2004**; **Howell, 2008**; **Johnson, 2016**; Kodjo, 2004; Monz, 2019; Paanaanen, 2013; Slade, 2002; Sullivan, 2015; Thomas, 2007	13/15	+ +
Racial/ethnic composition		**Cummings, 2014**; **Fitts, 2019**		0/2	0
County child population		**Hurlburt, 2004**		0/1	0
Social cohesion and control		van der Linden, 2003		0/1	0
*Public policy*					
Alternative quality contract		**Joyce, 2017**	**Joyce, 2017**	1/1	+
Fee-for-service plan compared to managed care plan		**Cook, 2004**; **Raghavan, 2006**	Brannan, 2005^e^; **Cook, 2004**; **Mandell, 2003**	3/4	+ +
Health insurance expansion	**Raghavan, 2006; Stein, 2012**	Hamersma, 2021; **Raghavan, 2006; Stein, 2012**	**Cidav, 2014**; Patrick, 1993; **Snowden, 2008**; **Stein, 2012**	4/6	+ +
Parity law		**Azrin, 2007**; **Barry, 2008**; **Block, 2020**; **Sen, 2018**	**Block, 2020**; **Li, 2020**; **Sen, 2018**; **Stuart, 2017**; **Walter, 2017**	5/7	+ +
Collaboration between organizations providing health services	**Hurlburt, 2004**	**Cole, 2019**	**Bai, 2009**; Grimes, 2018; **Rocks, 2020**	3/5	+ +
Coordination of care	Mann, 2021		**Witt, 2003**	1/2	?
Lockdown due to COVID-19	**Bakolis, 2021**; **Tromans, 2020**	**Bakolis, 2021; Tromans, 2020**	**Bakolis, 2021**	2/2	–
MH screening program		**Hacker, 2015; Hacker, 2017;** Sayal, 2010	Chisolm, 2009; **Hacker 2015; Hacker 2017;** Husky, 2011	4/5	+ +
Cambridge's police-mental health collaboration program		**Janopaul-Naylor, 2019**	**Janopaul-Naylor, 2019**	1/1	+
Large-scale mental health advocacy social media campaign			**Booth, 2018**	1/1	+
Telephone Support intervention		**Stevens, 2009**	McKay, 1998	1/2	?
Screening, brief intervention and referral to treatment (SBIRT)	**Sterling, 2019**	**Sterling, 2019**		1/1	–
*Macro-environmental level*					
Days of the week (mondays/tuesdays vs other)			Sobel, 1998	1/1	+
Holidays	Sobel, 1998			1/1	–
Lunar phase		Sobel, 1998		0/1	0
Seasons (fall vs other)			Sobel, 1998	1/1	+
Rainfall			Sobel, 1998	1/1	+
Snowfall			Sobel, 1998	1/1	+
Temperature (high/low vs normal)			Sobel, 1998	1/1	+
Displacement by hurricane	**Quast, 2018**	**Quast, 2018**		1/1	–

Bold: represents high-quality studies that adjusted for mental health problems. (a) *n* = number studies reporting significant association in the same direction; *N* = total number studies investigating association. If both negative and positive association are found, the highest number is reported. A significant association outweighs no association in the same study. b) For 3 studies: (0) no association, 0–33% of studies showed a significant association; (?) inconsistent association, 34–59% of studies reported significant associations; ( +) positive or (−) negative association, 60–100% of studies demonstrated significant associations. For 4 or more studies a summary of these associations is presented with (00), (??), (+ +), or (–), respectively. (c) Multiple studies from one database count as one in summarizing the evidence. d) Direction of association for a region of residence is less straightforward. e) Adjusted for mental health problems in part of the analyses but not all

### Organizational level

Twelve studies reported on 16 different determinants at the organizational level. Four of these studies were classified as high quality and adjusted for mental health problems. There was evidence for a positive association between having access to a school-based health center and mental health care utilization (5/6 studies). Of these six studies, only one was a high-quality well-adjusted study. All other 15 determinants had limited evidence (Table 3).

### Community level

Thirty-two studies reported on 9 determinants at the community level. Sixteen of these studies were classified as high quality and adjusted for mental health problems. Evidence for an association was found for the region of residence (8/10 studies). Six out of these ten studies were high quality and well-adjusted. Evidence for a positive association was found for high accessibility of services (5/8 studies; with 6/8 high-quality well-adjusted studies), living in an urban area (13/15 studies; with 7/15 high-quality well-adjusted studies) and living in an area with a high socio-economic status (5/9 studies; with 6/9 high-quality well-adjusted studies; multiple studies from one database count as one in summarizing the evidence). All other five determinants had limited evidence (Table 3).

### Public policy level

Thirty-six studies reported on 12 determinants at the public policy level. Twenty-seven of these studies were classified as high quality and adjusted for mental health problems. There was evidence for a positive association for implementing a parity law (i.e., treating the reimbursement of mental health care costs the same as other health care costs) (5/7 studies; with 7/7 high-quality well-adjusted studies), a mental health screening program (4/5 studies; with 2/5 high-quality well-adjusted studies), collaboration between health service providers (3/5 studies; with 4/5 high-quality well-adjusted studies), a fee-for-service plan compared to a managed care plan (3/4 studies; with 3/4 high-quality well-adjusted studies) and an expansion of health insurance (4/6 studies; with 4/6 high-quality well-adjusted studies). All other seven determinants had limited evidence (Table 3).

### Macro-environmental level

There were two studies examining eight determinants at the macro-environmental level, of which one was a high-quality well-adjusted study. All eight determinants had limited evidence.

## Discussion

This review aimed to summarize the evidence of contextual determinants associated with mental health care utilization in children and adolescents. In total, 45 determinants at the organizational, community, public policy and macro-environmental level were identified and for ten determinants evidence was found of an association with mental health care utilization in children and adolescents. Evidence for an association was found for having access to a school-based health center, region of residence, living in an urban area, living in an area with a high accessibility of mental health care, living in an area with a high socio-economic status, having a mental health parity law, a mental health screening program, collaboration between care provider organizations, fee-for-service plan (compared to a managed care plan) and extension of health insurance. These determinants were found in mostly high-quality well-adjusted studies, except for having access to a school-based health center, where only one study was of high quality and well-adjusted. For the other 35 identified determinants, the evidence was limited.

### Organizational level

At the organizational level, there was evidence for a positive association between having access to a school-based mental health center and mental health care utilization [67, 68, 75, 92, 106], but the quality and level of adjustment of the underlying studies were limited. Usually, children do not seek mental health care on their own but depend on caregivers, teachers or other adults to seek mental health care for them [109]. School-based health centers might play an important role in the access to mental health care since school health professionals are often the first professionals that are contacted when children have mental health problems [110]. Furthermore, school-based health center professionals receive specialized training for the identification of mental health problems in school-aged youth [75]. Children and adolescents in schools with a school-based health center benefit in terms of accessibility by receiving either on-site mental health screening and counseling services or by being referred to mental health services outside the school [17]. In addition, school-based health centers have in a previous systematic review also shown to be important for several educational and health-related outcomes [111]. School-based health centers might be a powerful tool to reach many children and adolescents at once and have several positive effects, as school is a place where early intervention can be facilitated [37].

In this review, only studies on school-related determinants were found at the organizational level. However, the organizational level also includes sports clubs or other organizations that are visited by children and adolescents [23]. These should be addressed in future research to get a more complete overview of determinants of mental health care use at the organizational level.

### Community level

Region of residence was associated with mental health care utilization [35, 41, 47, 52, 54, 64, 99, 105]. However, the direction of association is not straightforward for this determinant as this highly depends on which regions were compared with each other and which region served as the reference. As seen in this review, it is most likely that not the geographical location of the region itself is associated with mental health care utilization but the characteristics of that specific region. Evidence for a positive association was found for three of these area characteristics; a high area-level socio-economic status [35, 55, 57, 71, 76], high accessibility of health services [35, 38, 78, 88, 92] and an urban area [38, 44, 47, 50, 52, 64, 71, 77, 84, 85, 92, 100, 101]. These area characteristics might explain the association with the region of residence. In addition, the community level is a complex level where different factors can interfere with each other [112]. For example, living in an urban area is associated with higher accessibility of care, which can lead to higher care utilization [113]. Furthermore, the association between area-level socio-economic status and mental health care use can be dependent on the number of psychiatrists in the area [53, 114].

Although the evidence was limited, no association was found between area population characteristics and mental health care use, such as the racial/ethnic composition of the area [53, 57] and county child population (i.e., the number of children in an area) [65]. The role of ethnicity in predicting mental health care utilization might primarily be at the individual level rather than at the overall community level [57, 65].

### Public policy level

Determinants at the policy level such as parity laws and type of healthcare financing are mainly studied in the U.S.A., where there is no universal health coverage [115]. The most common insurance is private insurance, whereas public insurance is only for disadvantaged groups. There is also a group that is uninsured. In Europe, Canada and Australia, mental health care is covered by a national public health insurance program funded by taxes or government levies [115, 116]. This might explain why determinants at the policy level are less studied in these countries.

In this review, there was evidence of a positive association between parity laws and mental health care utilization [42, 79, 91, 98, 104]. Mental health parity requires insurance coverage for mental health conditions, including substance abuse disorder treatment to be equal to coverage for any other medical condition. A systematic review of policy levers reported that mental health parity increased access to children’s mental health services, by improved affordability [21]. Health insurance expansion also relates to improved affordability, for which evidence for a positive association was found in this review [49, 86, 93, 95].

Evidence for a positive association was also found in this review for the type of care financing (fee-for-service plan compared to a managed care plan). Under the fee-for-service model, health care providers receive payment for every delivered service, while under managed care the state pays a fixed amount per enrollee. The latter encourages more cost-effective service provision. In this review, children in a fee-for-service program were more likely to utilize mental health care compared to children in a managed care program [44, 52, 80]. However, such an association was not found in a study of children using welfare services who also have more extensive needs for mental health services [88].

Collaboration between mental health care providers was positively associated with mental health care utilization. One study found that the linkage between child welfare and mental health services led towards more care for children with the highest needs, but less mental health care in general [65]. Yet, the other studies showed that more collaboration and integrated care services led to more care utilization among children and adolescents with mental health problems.

The recognition of mental health problems is the first step in the decision to utilize mental health care [117]. Screening for mental health problems can facilitate this recognition and help professionals to refer children in need for care [118, 119]. Implementation of screening programs showed a positive association with mental health care utilization in various settings. The screening programs of the included studies were carried out by primary care providers, in waiting rooms of well-child clinics and at schools. One study did not find an association between screening and care utilization. A possible explanation is that they screened five-year-old children, which might be too young for an effective screening program as many mental health problems develop later in childhood [1].

### Macro-environmental level

For the determinants at the environmental level, evidence was limited. The identified determinants were derived from only two studies. A potential determinant at the macro-environmental level that is – in our opinion – missing in the current literature is the impact of the COVID-19 pandemic on mental health care use. The restrictions related to the pandemic have been studied at the policy level [39, 102], but not the effect of the pandemic itself.

### Strengths and limitations

To our knowledge, this was the first systematic review to investigate a broad range of contextual determinants associated with mental health care utilization in children and adolescents. In this review, a modified version of McLeroy’s ecological model was used to categorize the identified determinants, covering all contextual levels beyond the individual and interpersonal level [23]. Using another model might have led to a different grouping of the determinants. Nevertheless, placing a determinant within a certain level does not influence the level of evidence of that determinant. No restrictions were made on the type of study population or specific age groups, which increases the likelihood that all relevant studies on this topic were included. Furthermore, the search for this review was created by an experienced information specialist who has a PhD degree on literature retrieval in systematic reviews.

Nevertheless, some limitations of this review should also be acknowledged. First, a wide variety of mental health services and populations was included. Research suggests that determinants that are associated with service use may vary across types, amount of service and populations [22, 120], which might have increased the chance to find no or inconclusive associations in our study. Despite the diversity in services and populations, only studies from Western countries and among children under 21 years were included to increase the comparability among studies. Consequently, relevant articles including non-Western countries and people over 21 years might have been excluded.

Second, to assess the level of evidence, guidance for summarizing evidence across studies of different designs and populations is limited. Therefore, a pragmatic approach was chosen as developed and used by others in previously published research [32–34]. Choosing a more strict cut-off level (e.g. 67% instead of 60% of studies finding the same direction of an association) would lead to slightly different results. As can be seen in Table 3 this would lead to a different result for only one of the 10 determinant for which evidence was found, which is accessibility of services (inconsistent evidence instead of evidence for a positive association). Furthermore, the approach used for the assessment of the level of evidence does not allow to account for the quality of the studies. As most of the studies were of high quality this might not present a major problem. In the case of studies with limited quality or not well adjusted for mental health problems, this was clearly mentioned. For summarizing the evidence, determinants were grouped together, although the operationalization of determinants might have differed between studies. However, not grouping determinants together would have led to more determinants with limited evidence. Third, most of the included studies were considered as ‘high quality’. It might be that the cut-off for ‘high quality’ was too liberal as the QualSyst tool considers primarily the presence of certain components rather than the quality of these components. Further, the QualSyst tool might lack sensitivity to distinguish articles that are already of good quality from each other [121]. Yet, this tool has been used in many systematic reviews before and can be used for all types of study designs [28–31]. Most of the studies at the organizational level were not adjusted for mental health problems. Therefore, the findings should be interpreted with caution as the associations between the determinant and mental health care could be direct, but also indirect through mental health problems. Moreover, most of the studies in this review were conducted in the U.S.A. This decreases the external validity of the findings for countries in Europe and Oceania where the health care system is different [115, 116]. Last, the search strategy was restricted to English literature. Therefore, relevant studies carried out in different languages might have been missed.

### Implications and future research

Several implications can be drawn from this review. Determinants in contextual levels were identified, of which some can guide care providers and policy makers to improve the access to and provision of care. At the organizational level, evidence of an association between school-based health centers and mental health care utilization was found, although this should be interpreted with caution as most of the studies were of low quality and did not adjust for mental health problems. Our findings indicate that more research is necessary on school-based health centers and access to mental health services. If these centers are found to be important for access to mental health services, at the community level wider implementation of these centers could be warranted in areas that have lower mental health care use, such as areas with a low socioeconomic status, a low accessibility of services and rural areas. In addition, at the policy level, the implementation of mental health screening programs and expansion of mental health insurance coverage, including the implementation of parity laws are warranted as well as the encouragement of collaborations between organizations providing mental health care to increase mental health care utilization and improve access to care.

Future research is necessary, as for most of the identified determinants in this review limited evidence was available, meaning they were studied in three or less studies. To provide stronger evidence on contextual determinants of mental health care use, more research is needed on the understudied determinants. Especially at the organizational level, the number of studies was low. The only determinant with sufficient evidence was a school-based health center, which probably are typical for the U.S.A. Other forms of health service provision at schools that might be more common in countries outside the U.S.A. and their role in improving accessibility to mental health services for youth need further study. Moreover, at this organizational level, only school-related factors were studied, whereas other organizations, such as sports clubs or childcare/kindergarten facilities, can also play important roles in children’s access to care. Also, on the other contextual levels, studies on characteristics of care providers are lacking. Several examples are suggested by Stiffman et al. [109, 122], such as the level of training of the mental health professional, provider behavior, structure or culture of the organization providing care and collaboration between gateway providers. Because of the diversity in the measurement of determinants and mental health care use and a low number of studies on many determinants meta-analyses were not possible for this review. However, future studies should include meta-analyses on well-defined determinants.

Future research should also focus on an understudied age group, which is (early) childhood. Most of the included studies in this review were performed among adolescents. Another direction for future research is to focus on disentangling the mechanisms behind the association we found evidence for. For example, fee-for-service plans led to more care use as compared to managed care. It is important to understand the mechanism behind this association and whether this difference in financing system impacts the quality of care. Last, it is recommended to always adjust for mental health problems when studying mental health care use. In that way, the distinction can be made between determinants that are directly or indirectly related to mental health care use.

### Conclusion

This review identified ten contextual determinants that are positively associated with children’s and adolescents’ mental health care utilization; having access to a school-based health center, region of residence, living in an urban area, an area with a high accessibility of mental health care, an area with a high socio-economic status, implementing a mental health parity law, a mental health screening program, collaboration between organizations providing care, a fee-for-service plan (compared to a managed care plan) and extension of health insurance. Policymakers and care providers should be aware that contextual factors play a role in mental health care use by youth. Our findings indicate that addressing contextual factors is possible on organizational, community and public policy levels to improve access to and provision of care.

### Supplementary Information

Below is the link to the electronic supplementary material.

Supplementary file1 (PDF 813 KB)
