# Supplementary material for: Contextual determinants associated with children’s and adolescents’ mental health care utilization: a systematic review
Source: Eur Child Adolesc Psychiatry. 2022 Sep 21;33(7):2051–65. doi: 10.1007/s00787-022-02077-5 (PMC9490713; doi:10.1007/s00787-022-02077-5)
Supplement: Supplementary file 1 — Supplementary file1 (PDF 813 KB) [file 787_2022_2077_MOESM1_ESM.pdf]

Supplement 1

embase.com

('mental health care'/exp OR psychotherapy/de OR 'behavior therapy'/exp OR 'cognitive behavioral therapy'/exp OR 'cognitive therapy'/exp OR 'play therapy'/de OR 'family therapy'/exp OR psychiatrist/exp OR 'psychotherapist'/de OR 'parenting education'/de OR 'parent counseling'/de OR (((mental* OR mental-health OR psychosocial* OR psycho* OR psycho-education* OR psychiatr*) NEXT/1 (care OR healthcare OR service* OR treatment* OR consult OR consults OR unit OR units OR program* OR emergency-care OR health-care OR support-service* OR clinic OR clinics)) OR (behavioral NEXT/1 (service OR care OR healthcare)) OR youth-care OR (psychological* NEAR/3 (help OR service*)) OR psychotherap* OR psycho-therap* OR ((cogniti* OR behav* OR play OR family) NEAR/3 (interven* OR therap*)) OR psychiatrist* OR psychologist* OR pedagog* OR orthopedagog* OR (parenting* NEAR/3 (education OR support*)) OR (parent NEAR/3 counsel*)):ab,ti) AND (child/exp OR adolescent/exp OR adolescence/exp OR 'child behavior'/de OR 'child parent relation'/de OR pediatrics/exp OR childhood/exp OR 'child welfare'/de OR 'child abuse'/de OR 'child advocacy'/de OR 'child development'/de OR 'child growth'/de OR 'child health'/de OR 'child health care'/exp OR 'child care'/exp OR 'childhood disease'/exp OR 'child death'/de OR 'child psychiatry'/de OR 'child psychology'/de OR 'pediatric ward'/de OR 'pediatric hospital'/de OR (adolescen* OR preadolescen* OR infan* OR child* OR kid OR kids OR toddler* OR teen* OR boy* OR girl* OR minors OR underag* OR (under NEXT/1 (age* OR aging)) OR juvenil* OR youth* OR kindergar* OR puber* OR pubescen* OR prepubescen* OR prepubert* OR pediatric* OR paediatric* OR school* OR preschool* OR highschool*):ab,ti) AND ('controlled study'/exp OR 'control group'/exp OR 'cohort analysis'/exp OR 'population research'/exp OR 'Europe'/exp OR 'North America'/exp OR 'Australia and New Zealand'/exp OR (control* OR compar* OR cohort* OR population* OR national* OR international*):ab,ti) AND ('health care utilization'/exp OR (((utilizat* OR utilisat* OR enrol*) NEAR/6 (care OR healthcare OR service* OR source* OR resource* OR treatment* OR psychotherap* OR behav*-therap*)) OR (use-of NEXT/3 (care OR healthcare OR health-care OR service* OR source* OR resource* OR treatment* OR psychotherap* OR behav*-therap* OR cognit*-behav*-therap* OR speciali*-psychiat* OR speciali*-service*)) OR service-use):ab,ti) AND [English]/lim NOT ([Conference Abstract]/lim)

Medline Ovid

(exp Mental Health Services/ OR Psychotherapy/ OR exp Cognitive Therapy/ OR exp Behavior Therapy/ OR Play Therapy/ OR Family Therapy/ OR Psychiatry/ OR (((mental* OR mental-health OR psychosocial* OR psycho* OR psycho-education* OR psychiatr*) ADJ (care OR healthcare OR service* OR treatment* OR consult OR consults OR unit OR units OR program* OR emergency-care OR health-care OR support-service* OR clinic OR clinics)) OR (behavioral ADJ (service OR care OR healthcare)) OR youth-care OR (psychological* ADJ3 (help OR service*)) OR psychotherap* OR psycho-therap* OR ((cogniti* OR behav* OR play OR family) ADJ3 (interven* OR therap*)) OR psychiatrist* OR psychologist* OR pedagog* OR orthopedagog* OR (parenting* ADJ3 (education OR support*)) OR (parent ADJ3 counsel*)).ab,ti.) AND (exp Child/ OR exp Infant/ OR exp Adolescent/ OR exp "Child Behavior"/ OR exp "Parent Child Relations"/ OR exp "Pediatrics"/ OR exp "Child Welfare"/ OR "Child Development"/ OR exp "Child Health Services"/ OR exp "Child Care"/ OR "Child Rearing"/ OR exp "Child development Disorders, Pervasive"/ OR "Child Psychiatry"/ OR "Child Psychology"/ OR

"Hospitals, Pediatric"/ OR (adolescen* OR preadolescen* OR infan* OR child* OR kid OR kids OR toddler* OR teen* OR boy* OR girl* OR minors OR underag* OR (under ADJ (age* OR aging)) OR juvenil* OR youth* OR kindergar* OR puber* OR pubescen* OR prepubescen* OR prepubert* OR pediatric* OR paediatric* OR school* OR preschool* OR highschool*).ab,ti.) AND (controlled study/ OR Controlled Before-After Studies / OR Controlled Clinical Trial/ OR Control Groups / OR Cohort Studies / OR population research/ OR exp Europe/ OR exp North America/ OR exp Australia/ OR New Zealand/ OR (control* OR compar* OR cohort* OR population* OR national* OR international*).ab,ti.) AND ((((utilizat* OR utilisat* OR enrol*) ADJ6 (care OR healthcare OR service* OR source* OR resource* OR treatment* OR psychotherap* OR behav*-therap*)) OR ("use-of" ADJ (care OR healthcare OR health-care OR service* OR source* OR resource* OR treatment* OR psychotherap* OR behav*-therap* OR cognit*-behav*-therap* OR speciali*-psychiat* OR speciali*-service*)) OR service-use).ab,ti.) AND english.la.

PSycINFO Ovid

(exp Mental Health Services/ OR Psychotherapy/ OR exp Cognitive Therapy/ OR exp Behavior Therapy/ OR Play Therapy/ OR Family Therapy/ OR Psychiatry/ OR (((mental* OR mental-health OR psychosocial* OR psycho* OR psycho-education* OR psychiatr*) ADJ (care OR healthcare OR service* OR treatment* OR consult OR consults OR unit OR units OR program* OR emergency-care OR health-care OR support-service* OR clinic OR clinics)) OR (behavioral ADJ (service OR care OR healthcare)) OR youth-care OR (psychological* ADJ3 (help OR service*)) OR psychotherap* OR psycho-therap* OR ((cogniti* OR behav* OR play OR family) ADJ3 (interven* OR therap*)) OR psychiatrist* OR psychologist* OR pedagog* OR orthopedagog* OR (parenting* ADJ3 (education OR support*)) OR (parent ADJ3 counsel*)).ab,ti.) AND (100.ag. OR 200.ag. OR "Child Psychiatry"/ OR "Child Psychotherapy"/ OR "Child Psychology"/ OR Child Care/ OR (adolescen* OR preadolescen* OR infan* OR child* OR kid OR kids OR toddler* OR teen* OR boy* OR girl* OR minors OR underag* OR (under ADJ (age* OR aging)) OR juvenil* OR youth* OR kindergar* OR puber* OR pubescen* OR prepubescen* OR prepubert* OR pediatric* OR paediatric* OR school* OR preschool* OR highschool*).ab,ti.) AND (0300.md. OR 0430.md. OR 0450.md. OR Experiment Controls/ OR exp Europe/ OR (control* OR compar* OR cohort* OR population* OR national* OR international*).ab,ti.) AND ((((utilizat* OR utilisat* OR enrol*) ADJ6 (care OR healthcare OR service* OR source* OR resource* OR treatment* OR psychotherap* OR behav*-therap*)) OR ("use-of" ADJ (care OR healthcare OR health-care OR service* OR source* OR resource* OR treatment* OR psychotherap* OR behav*-therap* OR cognit*-behav*-therap* OR speciali*-psychiat* OR speciali*-service*)) OR service-use).ab,ti.) AND english.la.

Web of science

TS=(((((mental* OR mental-health OR psychosocial* OR psycho* OR psycho-education* OR psychiatr*) NEAR/1 (care OR healthcare OR service* OR treatment* OR consult OR consults OR unit OR units OR program* OR emergency-care OR health-care OR support-service* OR clinic OR clinics)) OR (behavioral NEAR/1 (service OR care OR healthcare)) OR youth-care OR (psychological* NEAR/2 (help OR service*)) OR psychotherap* OR psycho-therap* OR ((cogniti* OR behav* OR play OR family) NEAR/2 (interven* OR therap*)) OR psychiatrist* OR psychologist* OR pedagog* OR orthopedagog* OR (parenting* NEAR/2 (education OR support*)) OR (parent NEAR/2 counsel*))) AND ((adolescen* OR preadolescen* OR infan* OR child* OR kid OR kids OR toddler* OR teen* OR

boy* OR girl* OR minors OR underag* OR (under NEAR/1 (age* OR aging)) OR juvenil* OR youth* OR kindergar* OR puber* OR pubescen* OR prepubescen* OR prepubert* OR pediatric* OR paediatric* OR school* OR preschool* OR highschool*)) AND ((control* OR compar* OR cohort* OR population* OR national* OR international*)) AND ((((utilizat* OR utilisat* OR enrol*) NEAR/5 (care OR healthcare OR service* OR source* OR resource* OR treatment* OR psychotherap* OR "behav*-therap*")) OR ("use-of" NEAR/2 (care OR healthcare OR health-care OR service* OR source* OR resource* OR treatment* OR psychotherap* OR behav*-therap* OR cognit*-behav*-therap* OR speciali*-psychiat* OR speciali*-service*)) OR service-use))) AND DT=(article) AND LA=(english)

Cochrane CENTRAL

((((mental* OR mental next health OR psychosocial* OR psycho* OR psycho next education* OR psychiatr*) NEXT/1 (care OR healthcare OR service* OR treatment* OR consult OR consults OR unit OR units OR program* OR emergency next care OR health next care OR support next service* OR clinic OR clinics)) OR (behavioral NEXT/1 (service OR care OR healthcare)) OR youth next care OR (psychological* NEAR/3 (help OR service*)) OR psychotherap* OR psycho next therap* OR ((cogniti* OR behav* OR play OR family) NEAR/3 (interven* OR therap*)) OR psychiatrist* OR psychologist* OR pedagog* OR orthopedagog* OR (parenting* NEAR/3 (education OR support*)) OR (parent NEAR/3 counsel*)):ab,ti) AND ((adolescen* OR preadolescen* OR infan* OR child* OR kid OR kids OR toddler* OR teen* OR boy* OR girl* OR minors OR underag* OR (under NEXT/1 (age* OR aging)) OR juvenil* OR youth* OR kindergar* OR puber* OR pubescen* OR prepubescen* OR prepubert* OR pediatric* OR paediatric* OR school* OR preschool* OR highschool*):ab,ti) AND ((control* OR compar* OR cohort* OR population* OR national* OR international*):ab,ti) AND ((((utilizat* OR utilisat* OR enrol*) NEAR/6 (care OR healthcare OR service* OR source* OR resource* OR treatment* OR psychotherap* OR behav* next therap*)) OR (use next of NEXT/3 (care OR healthcare OR health next care OR service* OR source* OR resource* OR treatment* OR psychotherap* OR behav* next therap* OR cognit* next behav* next therap* OR speciali* next psychiat* OR speciali* next service*)) OR service next use):ab,ti)

Supplement 2

Table S1. Characteristics of the included studies (N=74)

**Study** *First author (year of publication)*	**Country**	**Study design**	**Number of participants**	**Database**	**Type of study population**	**Age group**	**Type of mental health care**	**Reporter mental health care utilization**	**Adjusted for mental health problems**
Abbas (2017)	Europe	Case-control	10,000-99,999	Health insurance funds data	With mental health problems/care	More	Outpatient	Administrative data	Yes
Atkins (2015)	Northern America	Randomized study	100-999	NA	Low income and mental health problems/care	Childhood	Outpatient	Administrative data	No
Azrin (2007)	Northern America	Quasi-experimental	100,000-999,999	NA	General population	More	Outpatient, inpatient and medication use	Administrative data	Yes
Bai (2009)	Northern America	Cohort study	1,000-9,999	Child Protective Services (CPS) cohort of the National Survey of Child and Adolescent Well-Being (NSCAW)	With mental health problems/care	Childhood	Outpatient	Caregiver	Yes
Bakolis (2021)	Europe	Quasi-experimental	≥1,000,000	National Institute for Health Research (NIHR) Mental Health Translational Research Collaboration	General population	More	Outpatient	Administrative data	Yes
Barry (2008)	Northern America	Quasi-experimental	10,000-99,999	National Survey of America's Families (NSAF)	General population	More	Outpatient	Caregiver	Yes
Bird (2008)	Northern America	Cohort study	1,000-9,999	National Institute of Mental Health (NIMH)	General population	Childhood	Outpatient, inpatient and medication use	Caregiver	Yes
Block (2020)	Northern America	Quasi-experimental	≥1,000,000	NA	General population	More	Outpatient and inpatient	Administrative data	Yes
Booth (2018)	Northern America	Quasi-experimental	100,000-999,999	Registered Persons Database (RPDB)	With mental health problems/care	More	Outpatient	Administrative data	Yes
Brannan (2005)	Northern America	Cohort study	100-999	Impact of Medicaid Managed Care (IMMC)	Low income	More	Outpatient and inpatient	Caregiver	In part of the analyses
Britto (2001)	Northern America	Quasi-experimental	1,000-9,999	NA	General population	Adolescence	Outpatient	Self-report	No
Brown (2014)	Northern America	Cross-sectional	1,000-9,999	National Health Interview Survey (NHIS)	With mental health problems/care	More	Unknown	Caregiver	No
Bryson (2015)	Northern America	Cross-sectional	10,000-99,999	Kansas Medicaid Management Information System (NMIS)	Low income and mental health problems/care	More	Inpatient	Administrative data	Yes
Chisolm (2009)	Northern America	Randomized study	1,000-9,999	NA	General population	Adolescence	Outpatient	Administrative data	Yes

Cidav (2014)	Northern America	Cross-sectional	10,000-99,999	Medicaid Analytic Extract (MAX) data files	With mental health problems/care	More	Outpatient and inpatient	Administrative data	Yes
Cohen (1993)	Northern America	Cohort study	100-999	Children in the Community Study	General population	adolescence	Outpatient	Caregiver	Yes
Cole (2019)	Northern America	Quasi-experimental	1,000-9,999	NA	Low income	Adolescence	Outpatient and inpatient	Administrative data	Yes
Cook (2004)	Northern America	Cohort study	1,000-9,999	Substance Abuse and Mental health Service Administration-funded Managed Behavioral Health Care in the Public Sector Study	Low income and mental health problems/care	More	Outpatient and inpatient	Caregiver	Yes
Cummings (2014)	Northern America	Cohort study	1,000-9,999	National Longitudinal Study of Adolescent Health (first two waves)	With mental health problems/care	Adolescence	Outpatient and medication use	Self-report	Yes
Davila (2020)	Northern America	Cohort study	1,000-9,999	Multiple registries	Other	More	Outpatient and inpatient	Administrative data	No
Efron (2019)	Oceania	Cohort study	100-999	Children's Attention Project	With mental health problems/care	Childhood	Medication use	Caregiver	Yes
Finnvold (2019)	Europe	Cross-sectional	100,000-999,999	Two large register datasets	General population	More	Outpatient and inpatient	Administrative data	No
Fitts (2019)	Northern America	Cohort study	1,000-9,999	Longitudinal outcome study of the National Evaluation of the CMHI	With mental health problems/care	More	Outpatient	Caregiver	Yes
Green (2013)	Northern America	Cohort study	1,000-9,999	National Comorbidity Survey Adolescent Supplement (NCS-A)	General population	Adolescence	Outpatient, inpatient and medication use	Self-report + caregiver	Yes
Grimes (2018)	Northern America	Quasi-experimental	100-999	NA	With mental health problems/care	More	Outpatient and medication use	Administrative data	Yes
Hacker (2017)	Northern America	Quasi-experimental	≥1,000,000	NA	Low income	More	Outpatient, inpatient and medication use	Administrative data	Yes
Hacker (2015)	Northern America	Quasi-experimental	10,000-99,999	Cambridge Health Alliance data warehouse	General population	More	Outpatient and inpatient	Administrative data	Yes
Halladay (2020)	Northern America	Cross-sectional	10,000-99,999	School Mental Health Survey (SMHS)	General population	Adolescence	Outpatient and inpatient	Self-report	Yes
Hamersma (2021)	Northern America	Cohort study	10,000-99,999	National Survey of America's Families (NSAF)	Low income	More	Outpatient	Caregiver	No
Howell (2008)	Northern America	Cross-sectional	10,000-99,999	National Survey of America's Families (NSAF)	Low income	More	Outpatient	Caregiver	Yes
Hurlburt (2004)	Northern America	Cohort study	1,000-9,999	National Survey of Child and Adolescent Well-being (NSCAW)	involved with child welfare	More	Outpatient	Caregiver	Yes
Husky (2011)	Northern America	Randomized study	100-999	NA	General population	Adolescence	Outpatient	Administrative data	No
Hussaini (2021)	Northern America	Cohort study	10,000-99,999	NA	General population	Adolescence	Unknown	Administrative data	No

Hutchinson (2012)	Northern America	Quasi-experimental	1,000-9,999	School Health Connection Survey	General population	Adolescence	Outpatient	Self-report	Yes
Ivert (2013)	Europe	Cross-sectional	10,000-99,999	Longitudinal Multilevel Analysis in Scania (LOMAS)	General population	Adolescence	Outpatient, inpatient and medication use	Administrative data	No
Janopaul-Naylor (2019)	Northern America	Quasi-experimental	100-999	NA	Other	More	Outpatient, inpatient and medication use	Administrative data	Yes
Johnson (2016)	Oceania	Cross-sectional	100-999	Young Minds Matter	With mental health problems/care	More	Outpatient and inpatient	Self-report + caregiver	Yes
Joyce (2017)	Northern America	Quasi-experimental	Other	NA	With mental health problems/care	More	Outpatient, inpatient and medication use	Administrative data	Yes
Kang-Yi (2013)	Northern America	Cohort study	100-999	NA	Low income	More	Inpatient	Administrative data	Yes
Kaplan (1999)	Northern America	Cohort study	1,000-9,999	NA	Low income	Childhood	Outpatient	Caregiver	No
Kaplan (1998)	Northern America	Cohort study	100-999	NA	General population	Adolescence	Unknown	Administrative data	No
Kim (2018)	Northern America	Cohort study	1,000-9,999	National Survey of Child and Adolescent Well-being (NSCAW)	Involved with child welfare	Adolescence	Outpatient	Caregiver	No
Kodjo (2004)	Northern America	Cohort study	1,000-9,999	National Longitudinal Study of Adolescent Health (first two waves)	With mental health problems/care	Adolescence	Outpatient	Self-report	No
Kovess-Masfety (2017)	Europe	Cross-sectional	1,000-9,999	School Children Mental Health in Europe Project	General population	Childhood	Outpatient and inpatient	Caregiver	Yes
Li (2020)	Northern America	Quasi-experimental	10,000-99,999	National Survey of Children's Health	General population	More	Unknown	Caregiver	Yes
Mandell (2003)	Northern America	Cross-sectional	1,000-9,999	NA	Low income	More	Unknown	Administrative data+ caregiver	Yes
Mann (2021)	Northern America	Cohort study	100-	NA	With mental health problems/care	More	Outpatient and inpatient	Administrative data	No
McKay (1998)	Northern America	Cohort study	100-999	NA	General population	More	Unknown	Agency's research staff	No
Mendenhall (2012)	Northern America	Randomized study	100-999	Multi-Family Psychoeducational Psychotherapy study	With mental health problems/care	Childhood	Unknown	Caregiver	Yes
Monz (2019)	Northern America	Cross-sectional	1,000-9,999	Simons Foundation Powering Autism Research for Knowledge (SPARK cohort)	With mental health problems/care	Adolescence	Outpatient and inpatient	Caregiver	No
Paananen (2013)	Europe	Cohort study	10,000-99,999	NA	General population	More	Outpatient and inpatient	Administrative data	No
Patrick (1993)	Northern America	Cohort study	100,000-999,999	NA	General population	More	Inpatient	Administrative data	No

Quast (2018)	Northern America	Case-control	10,000-99,999	NA	Low income	More	Outpatient and inpatient	Administrative data	Yes
Raghavan (2006)	Northern America	Cohort study	1,000-9,999	National Survey of Child and Adolescent Well-being (NSCAW)	involved with child welfare	More	Outpatient and inpatient	Caregiver	Yes
Rocks (2020)	Europe	Quasi-experimental	Other	NA	With mental health problems/care	more	Unknown	Administrative data	Yes
Sayal (2010)	Europe	Randomized study	100-999	NA	With mental health problems/care	Childhood	Outpatient and inpatient	Caregiver	Yes
Sen (2018)	Northern America	Cohort study	≥1,000,000	NA	General population	More	Outpatient and inpatient	Administrative data	Yes
Slade (2002)	Northern America	Cross-sectional	10,000-99,999	National Longitudinal Study of Adolescent Health (first two waves)	General population	More	Outpatient	Self-report	Yes
Snowden (2008)	Northern America	Quasi-experimental	Other	NA	Low income	More	Outpatient and inpatient	Administrative data	Yes
Sobel (1998)	Northern America	Cross-sectional	1,000-9,999	NA	General population	More	Inpatient	Administrative data	No
Stein (2012)	Northern America	Cohort study	10,000-99,999	NA	Low income and mental health problems/care	More	Outpatient, inpatient and medication use	Administrative data	Yes
Sterling (2019)	Northern America	Cohort study	1,000-9,999	NA	With mental health problems/care	Adolescence	Outpatient and inpatient	Administrative data	Yes
Stevens (2009)	Northern America	Cohort study	100-999	NA	With mental health problems/care	Adolescence	Outpatient and inpatient	Self-report	Yes
Stuart (2017)	Northern America	Quasi-experimental	10,000-99,999	NA	With mental health problems/care	More	Outpatient, inpatient and medication use	Administrative data	Yes
Sturm (2003)	Northern America	Cross-sectional	10,000-99,999	National Survey of America's Families (NSAF)	Low income	More	Outpatient and inpatient	Caregiver	No
Sullivan (2015)	Northern America	Cross-sectional	1,000-9,999	National Longitudinal Transition Study 2	With disabilities	Adolescence	Medication use	Caregiver	No
Thomas (2007)	Northern America	Cross-sectional	100-999	NA	With mental health problems/care	Childhood	Outpatient, inpatient and medication use	Caregiver	Yes
Tromans (2020)	Europe	Cohort study	100,000-999,999	NA	General population	More	Unknown	Administrative data	Yes
Van der Linden (2003)	Europe	Case-control	100-999	NA	General population	Childhood	Unknown	Administrative data	No
Walter (2017)	Northern America	Cohort study	≥1,000,000	NA	With mental health problems/care	More	Outpatient	Administrative data	Yes
Waxmonsky (2019)	Northern America	Cohort study	100,000-999,999	NA	With mental health problems/care	More	Outpatient and medication use	Administrative data	Yes
Williams (2015)	Northern America	Cross-sectional	1,000-9,999	National Longitudinal Study of Adolescent Health	With mental health problems/care	Adolescence	Outpatient and inpatient	Self-report	No

Witt (2003)	Northern America	Cross-sectional	1,000-9,999	National Health Interview Survey Disability Supplements	With disabilities	More	Outpatient and inpatient	Caregiver	Yes
Zablotsky (2019)	Northern America	Cross-sectional	100-999	National Health Interview Survey	With mental health problems/care	More	Outpatient and inpatient	Caregiver	No

Supplement 3

Table S2. Quality assessment of the included studies using the QualSyst Tool by Kmet et al. (2004) with scores per item

**Study** *First author (year of publication)*	**1. Research question**	**2. Study design**	**3. Subject and variable selection**	**4. Subject characteristics**	**5. Rando-mization described**	**6. Blinding investigators**	**7. Blinding subject**	**8. Exposures and outcome**	**9. Sample size**	**10. Analytic methods**	**11. Estimate of variance**	**12. Confounding**	**13. Results in sufficient detail**	**14. Conclu-sions support results**	**Quality score**	**Level of quality**
Abbas (2017)	2	2	2	1	NA	NA	NA	2	2	2	2	2	2	2	0,95	High
Atkins (2015)	2	2	1	2	1	2	NA	2	1	2	2	2	2	1	0,85	High
Azrin (2007)	2	2	2	1	NA	NA	NA	2	2	2	2	1	2	2	0,91	High
Bai (2009)	2	1	2	2	NA	NA	NA	2	2	2	2	2	2	2	0,95	High
Bakolis (2021)	2	2	1	NA	NA	NA	NA	1	2	2	2	2	2	2	0,90	High
Barry (2008)	2	1	1	2	NA	NA	NA	2	2	2	2	2	2	2	0,91	High
Bird (2008)	2	2	1	0	NA	NA	NA	2	2	2	2	2	2	2	0,86	High
Block (2020)	2	2	2	2	NA	NA	NA	2	2	2	2	1	2	2	0,95	High
Booth (2018)	2	2	2	2	NA	NA	NA	2	2	2	2	1	2	2	0,95	High
Brannan (2005)	1	1	1	2	NA	NA	NA	2	2	2	2	2	2	2	0,86	High
Britto (2001)	2	2	1	2	0	NA	NA	1	2	1	0	2	2	1	0,67	Medium
Brown (2014)	1	0	1	0	NA	NA	NA	2	2	2	0	2	2	2	0,64	Medium
Bryson (2015)	1	2	2	2	NA	NA	NA	1	2	2	2	2	2	2	0,91	High
Chisolm (2009)	2	1	0	2	1	0	NA	2	2	2	2	1	1	2	0,69	Medium
Cidav (2014)	2	1	2	1	NA	NA	NA	2	2	2	0	2	2	2	0,82	High
Cohen (1993)	2	2	2	2	NA	NA	NA	2	2	2	2	1	2	2	0,95	High
Cole (2019)	2	1	2	1	NA	NA	NA	2	2	2	2	1	2	2	0,86	High
Cook (2004)	2	2	2	2	NA	NA	NA	2	2	2	0	2	2	2	0,91	High
Cummings (2014)	1	1	1	2	NA	NA	NA	2	2	2	2	2	2	2	0,86	High
Davila (2020)	2	2	2	1	NA	NA	NA	2	2	2	2	2	2	2	0,95	High
Efron (2019)	2	2	1	2	NA	NA	NA	2	1	2	2	2	2	2	0,91	High
Finnvold (2019)	1	1	2	2	NA	NA	NA	2	2	2	0	2	2	1	0,77	Medium
Fitts (2019)	1	1	1	2	NA	NA	NA	2	2	2	2	2	2	2	0,86	High
Green (2013)	2	1	2	0	NA	NA	NA	2	2	2	2	2	2	2	0,86	High
Grimes (2018)	2	2	1	2	0	NA	NA	1	1	2	2	2	2	2	0,79	Medium
Hacker (2017)	2	2	1	2	NA	NA	NA	2	2	2	2	2	2	2	0,95	High
Hacker (2015)	2	2	2	2	NA	NA	NA	2	2	2	2	2	2	2	1,00	High
Halladay (2020)	2	2	1	2	NA	NA	NA	2	2	2	2	2	2	2	0,95	High
Hamersma (2021)	1	1	1	0	NA	NA	NA	2	2	2	2	2	1	2	0,73	Medium
Howell (2008)	2	1	1	1	NA	NA	NA	2	2	2	2	2	1	2	0,82	High
Hurlburt (2004)	2	1	2	2	NA	NA	NA	2	2	2	2	2	1	2	0,91	High

Husky (2011)	2	2	2	2	1	0	NA	2	1	2	2	2	1	1	0,77	Medium
Hussaini (2021)	2	2	1	2	NA	NA	NA	2	2	2	2	2	2	2	0,95	High
Hutchinson (2012)	2	2	1	2	NA	NA	NA	1	2	2	2	1	2	2	0,86	High
Ivert (2013)	2	1	2	2	NA	NA	NA	2	2	2	2	2	2	2	0,95	High
Janopaul-Naylor (2019)	1	2	1	2	NA	NA	NA	2	2	2	0	2	2	2	0,82	High
Johnson (2016)	2	1	1	1	NA	NA	NA	2	2	2	2	2	1	2	0,82	High
Joyce (2017)	2	2	2	0	NA	NA	NA	2	2	2	2	2	2	2	0,91	High
Kang-Yi (2013)	2	1	1	2	NA	NA	NA	2	2	2	2	2	2	2	0,91	High
Kaplan (1999)	1	1	1	1	NA	NA	NA	2	2	2	2	2	2	2	0,82	High
Kaplan (1998)	2	2	2	1	NA	NA	NA	2	2	1	NA	1	2	2	0,85	High
Kim (2018)	2	2	2	2	NA	NA	NA	2	1	2	2	2	2	1	0,91	High
Kodjo (2004)	2	2	1	2	NA	NA	NA	2	2	2	2	2	2	2	0,95	High
Kovess-Masfety (2017)	2	2	1	2	NA	NA	NA	2	2	2	2	1	1	2	0,86	High
Li (2020)	1	2	1	2	NA	NA	NA	1	2	2	2	2	2	2	0,86	High
Mandell (2003)	2	1	1	2	NA	NA	NA	2	2	2	2	2	2	2	0,91	High
Mann (2021)	2	1	1	2	NA	NA	NA	2	1	2	2	1	1	2	0,77	Medium
McKay (1998)	2	1	1	2	1	0	NA	2	1	1	NA	NA	1	2	0,64	Medium
Mendenhall (2012)	2	2	1	1	NA	NA	NA	1	1	2	0	2	2	2	0,73	Medium
Monz (2019)	2	1	1	2	NA	NA	NA	2	2	2	2	1	2	2	0,86	High
Paananen (2013)	2	2	1	2	NA	NA	NA	2	2	2	2	2	2	2	0,95	High
Patrick (1993)	2	1	2	2	NA	NA	NA	2	2	2	2	2	2	2	0,95	High
Quast (2018)	2	1	2	2	NA	NA	NA	2	2	2	0	1	2	2	0,82	High
Raghavan (2006)	2	2	2	2	NA	NA	NA	2	2	2	2	2	2	2	1,00	High
Rocks (2020)	2	2	2	2	NA	NA	NA	2	2	2	2	2	2	2	1,00	High
Sayal (2010)	2	2	1	0	1	0	NA	2	2	2	2	2	2	2	0,77	Medium
Sen (2018)	2	1	2	0	NA	NA	NA	2	2	2	2	2	2	2	0,86	High
Slade (2002)	1	1	1	2	NA	NA	NA	2	2	2	0	2	2	2	0,77	Medium
Snowden (2008)	2	2	2	NA	NA	NA	NA	2	2	2	2	2	2	2	1,00	High
Sobel (1998)	1	1	2	1	NA	NA	NA	1	2	1	NA	NA	2	2	0,72	Medium
Stein (2012)	2	1	2	2	NA	NA	NA	2	2	2	0	2	2	2	0,86	High
Sterling (2019)	1	2	2	2	1	0	NA	1	2	2	2	2	2	2	0,81	High
Stevens (2009)	2	1	1	2	2	2	NA	2	2	2	0	2	2	2	0,85	High
Stuart (2017)	2	2	2	2	NA	NA	NA	2	2	2	2	1	2	2	0,95	High
Sturm (2003)	2	1	1	0	NA	NA	NA	1	2	2	0	2	2	2	0,68	Medium
Sullivan (2015)	1	1	2	2	NA	NA	NA	2	2	2	2	2	2	2	0,91	High
Thomas (2007)	1	1	1	2	NA	NA	NA	2	1	2	2	2	1	2	0,77	Medium
Tromans (2020)	2	1	2	NA	NA	NA	NA	2	2	1	2	1	2	2	0,85	High
Van der Linden (2003)	2	1	1	2	NA	NA	NA	2	2	2	2	2	2	2	0,91	High

Walter (2017)	2	1	2	2	NA	NA	NA	2	2	2	2	2	2	2	0,95	High
Waxmonsky (2019)	1	2	2	2	NA	NA	NA	2	2	2	2	2	2	2	0,95	High
Williams (2015)	2	2	1	2	NA	NA	NA	2	2	2	2	2	2	2	0,95	High
Witt (2003)	1	1	1	2	NA	NA	NA	2	2	2	2	2	2	2	0,86	High
Zablotsky (2019)	2	1	1	2	NA	NA	NA	2	2	2	2	2	2	2	0,91	High

Each item was scored depending on to what degree the criterion was met: yes = 2 points, partial = 1 point, no = 0. For more information on the items, see Kmet et al. (2004).
